# Integrating art therapy and technology in neurorehabilitation: a scoping review

**DOI:** 10.3389/fneur.2026.1727248

**Published:** 2026-02-02

**Authors:** Marta Albani-Rocchetti, Anna Roman, Sara Federico, Martina Regazzetti, Luisa Cacciante, Błażej Cieślik, Adam Wrzeciono, Joanna Szczepańska-Gieracha, Roman Nowobilski, Pawel Kiper

**Affiliations:** 1 Healthcare Innovation Technology Lab, IRCCS San Camillo Hospital, Venice, Italy; 2 Collegium Medicum, Jan Dlugosz University in Czestochowa, Czestochowa, Poland; 3 WSB Merito University in Gdańsk, Gdańsk, Poland; 4 Faculty of Health Sciences, Jagiellonian University Medical College, Kraków, Poland

**Keywords:** art therapy, dance therapy, digital rehabilitation, music therapy, Parkinson’s disease, stroke, virtual reality

## Abstract

**Background:**

Art therapy is emerging as a promising adjunct to neurorehabilitation, giving creative engagement to improve motor, cognitive, and emotional outcomes. Digital technologies such as virtual reality (VR), augmented reality (AR), exergames, and sensor-based systems enable immersive and interactive therapeutic experiences, potentially enhancing rehabilitation effectiveness. This scoping review systematically evaluates the impact of technology-assisted art therapy on neurological rehabilitation and to identify effective intervention types.

**Methods:**

A systematic search was conducted in PubMed, Cochrane, Web of Science, and Embase following PRISMA-ScR and JBI guidelines. Studies were included if they involved adults with neurological conditions receiving technology-supported art therapy and reported motor, cognitive, or emotional outcomes.

**Results:**

Of 584 records screened, 19 studies were included. Interventions comprised dance therapy, music therapy, and visual art therapy supported by VR platforms, tablet-based applications, serious games, and motion-tracking systems. Reported benefits included improvements in motor function, attention and executive function, emotional well-being, and therapy engagement. However, most studies were small-scale, with heterogeneous methodologies and limited follow-up periods.

**Conclusion:**

Technology-enhanced art therapy appears to be a promising approach in neurorehabilitation, offering personalized, engaging, and potentially effective interventions. Further high-quality randomized controlled trials with standardized outcome measures are needed to confirm these findings and guide clinical application.

## 1 Introduction

In recent years, the scientific and medical communities have increasingly recognized the fundamental role of art in health promotion, preventing illness, and supporting treatment (1). As emphasized by the World Health Organization (WHO), the arts, in all their forms, can have a significant impact on physical, psychological, and social well-being (1). The American Art Therapy Association (AATA) in About Art Therapy (2018), defined art therapy as a therapeutic modality used over ongoing sessions to improve cognitive and sensorimotor functions, foster self-esteem and self-awareness, cultivate emotional resilience, promote insight, enhance social skills, reduce and resolve conflicts and distress and advance societal and ecological change (2). Art therapy is not merely esthetic; it becomes a transformative and expressive channel that fosters healing, self-awareness, and improved quality of life (3).

One particularly promising field for art therapy is neurorehabilitation, the recovery of brain functions impaired by trauma, stroke, or neurological disease (3–5). Creativity represents a core construct underlying art therapy interventions and is defined as “the interaction among aptitude, process, and environment by which an individual or group produces a perceptible product that is both novel and useful within a social context” (6). Creativity arises from coordinated interactions between the default mode and executive control networks, enabling the generation and regulation of novel ideas through a balance of spontaneous and controlled cognitive processes (7, 8). Neurological disorders may alter these networks in heterogeneous ways, leading not only to impairments but also, in some cases, to paradoxical enhancements of creativity (9). Recent neuroimaging evidence demonstrates that brain regions activated during creative tasks map onto a distributed brain circuit characterized by negative functional connectivity with the right frontal pole, reflecting reduced top-down inhibitory control (10). Damage to this creativity-related circuit due to focal brain lesions or neurodegenerative diseases has been associated with both decreases and paradoxical increases in creativity, supporting the notion that creative processes may be preserved, reshaped, or even facilitated following neurological damage (10). Chen et al. showed that all three creative domains (drawing, music, and literary creativity) activated the pre-supplementary motor area (pre-SMA), the left dorsolateral prefrontal cortex (DLPFC), and the right inferior frontal gyrus (IFG), indicating that these brain regions play a general role in artistic creativity. In addition, some areas appeared to be more specialized for particular forms of creativity (11, 20). However, Abraham showed that common neuroimaging paradigms used to study creativity have major methodological limits, making results difficult to interpret and highlighting the need for more appropriate and ecologically valid experimental designs to better understand the neuroscience of creativity (12). Within this framework, art therapy may leverage preserved or reconfigured creative networks to support rehabilitation processes in individuals with neurological disorders.

Research suggests that integrating art therapy into conventional treatment plans could lead to improved emotional well-being and social engagement for patients with neurodegenerative diseases (13). Artistic engagement can stimulate neuroplasticity, activating motor and sensory brain areas and promoting use-dependent learning, a recovery process based on repeated activation and stimulation of affected functions (8, 9, 14). The concept of flow, a psychological condition in which individuals become fully immersed in an activity, losing track of time and their surroundings rapresents a key mechanism which creative engagement can help stimulate brain activity and facilitate functional recovery, regardless of age (15).

With technological advances, new possibilities are emerging in the field of art therapy (16). In particular, Virtual Reality (VR) is gaining attention as an innovative tool capable of offering immersive and interactive artistic experiences. VR allows patients to explore rich, stimulating, and customizable visual environments, providing a safe and controlled space for artistic expression. As noted by Aldridge and Bethel, VR-based art therapy can offer sensory stimulation and measurable performance outcomes, both of which are essential in neurorehabilitation, especially for individuals recovering from brain injuries (3). Other emerging technologies include Augmented Reality (AR), which overlays digital content onto the real world for real-time, context-sensitive feedback (17), and game-based interventions, such as exergames (video games requiring physical movement) (18) and serious games (designed for therapeutic or educational purposes) (19). Digital tools including painting software, animation platforms, and sensorized systems further enhance expressive possibilities, increasing accessibility, engagement, and interactivity (16). Additionally, the rise of telehealth has helped eliminate geographic barriers, making art therapy available remotely (21). Despite these promising innovations, many questions remain unanswered (22).

There is currently no comprehensive synthesis of how digital and immersive tools—including VR, AR, exergames, and digital art platforms—are implemented in neurological rehabilitation, what outcomes are observed, and what challenges arise in real-world practice. Evidence remains limited regarding the implementation, clinical outcomes, and sustainability of technology-assisted art therapy.

This scoping review aims to systematically map and analyze the use of art therapy integrated with digital and immersive technologies in neurorehabilitation. The goal is to understand how various creative modalities are supported and enhanced through technological tools, evaluating their practical applications, therapeutic potential, and related challenges. The review seeks to provide a comprehensive overview of existing evidence to identify current clinical practices and highlight areas requiring further research, thereby promoting more effective and accessible use of art therapy integrated with techonologies in neurological populations. In depth, in this review, the term “art therapy” encompasses a wide range of creative modalities (23), including: Performing arts (e.g., music, dance); Visual arts (e.g., painting, sculpture); Literature (e.g., writing, reading); Digital arts (e.g., animation, digital painting). These categories will be analyzed in the context of their integration with digital technologies and their therapeutic impact on neurological populations.

## 2 Materials and methods

This protocol followed the “JBI Manual for Evidence Synthesis” Chapter 10.2 “Development of a scoping review protocol,” and reported using “PRISMA extension for scoping reviews (PRISMA–ScR)” (24). The review protocol was registered prospectively with the Open Science Framework on 19 May of 2025.^1^

### 2.1 Eligibility criteria

The eligibility criteria for study inclusion were defined following the Population, Concept, and Context (PCC) framework, in alignment with the previously stated research questions. To be included in the review, studies had to involve adult participants diagnosed with neurological diseases, including but not limited to stroke, spinal cord injury, multiple sclerosis, or Parkinson’s disease. These conditions were selected as they are known to affect the nervous system and may result in impairments of motor function, sensation, and/or cognition, which are central aspects in rehabilitation. The concept of interest was the application of art therapy as a rehabilitation strategy. In this review, art therapy was considered in a broad sense, encompassing: Performing arts (e.g., music, dance), Visual arts (e.g., painting, sculpture), Literature-based activities (e.g., reading, creative writing) and Digital arts (e.g., digital drawing, animation). To be eligible, the art therapy intervention must have been developed and implemented through a technological system, such as virtual reality and delivered within a rehabilitation setting.

Studies also needed to report on outcome measures evaluating the impact or effectiveness of the intervention. Further inclusion criteria were: Peer-reviewed publications, written in English, to ensure interpretability and accuracy of data extraction. No publication date restriction was applied during the selection process. This decision was made due to the limited availability of studies specifically addressing the intersection of art therapy, neurological rehabilitation, and technological implementation. Studies were excluded if they did not utilize a technological system in delivering art therapy, were conducted outside a rehabilitation context (e.g., educational or general community settings), focused on populations other than adults with neurological conditions, lacked outcome measures, or addressed only the psychological or recreational aspects of art therapy without a rehabilitative goal. In addition, systematic reviews, meta-analyses, and other forms of secondary research were not included.

### 2.2 Information sources and search strategies

The search process began with the identification of relevant keywords and controlled vocabulary terms (e.g., MeSH terms) based on the research questions and PCC framework. After selecting the appropriate terms, search strings (Appendix 1) were constructed using Boolean operators and adapted for each database. A comprehensive search was conducted in the following electronic databases: PudMed, Web of Science, Cochrane Library and Embase. Search results were exported into Rayyan QCRI, a web-based platform for systematic review screening. Duplicate records were automatically identified and removed within the platform. Title and abstract screening was independently conducted by two reviewers using Rayyan’s blind mode. Any disagreements were resolved through discussion with a third reviewer after unblinding. Full text screening was done by 4 reviewers (M. R; A. W, M. A. R, A. R).

### 2.3 Data charting and data items

A data-charting form was jointly developed by four reviewers to determine which variables to extract. The four reviewers independently charted the data, discussed the results and continuously updated the data-charting form in an iterative process. The following information were collected from each study: Author(s), year of publication, Study type and design (e.g., RCT, qualitative study, review), Aim of the study, Population characteristics (e.g., condition, sample size), Type of art therapy intervention (e.g., music, visual arts, literature), Technology used (e.g., VR, digital platforms), Duration/Dose of the Intervention, Outcomes measured, Key findings and conclusions.

### 2.4 Synthesis of results

The studies were grouped based on the type of art therapy intervention examined and type of pathology. The summary table was developed to synthesize key information from the included studies and was used to address the following research questions:

- What are the main art therapy techniques applied in VR (e.g., drawing, music, etc.)?
- What types of technology are used to conduct art therapy in neurorehabilitation?
- What are the primary outcomes studied in the research on virtual art therapy in neurorehabilitation?
- What are the positive and negative factors of virtual art therapy?

## 3 Results

### 3.1 Selection of source of evidence

**Figure 1:**
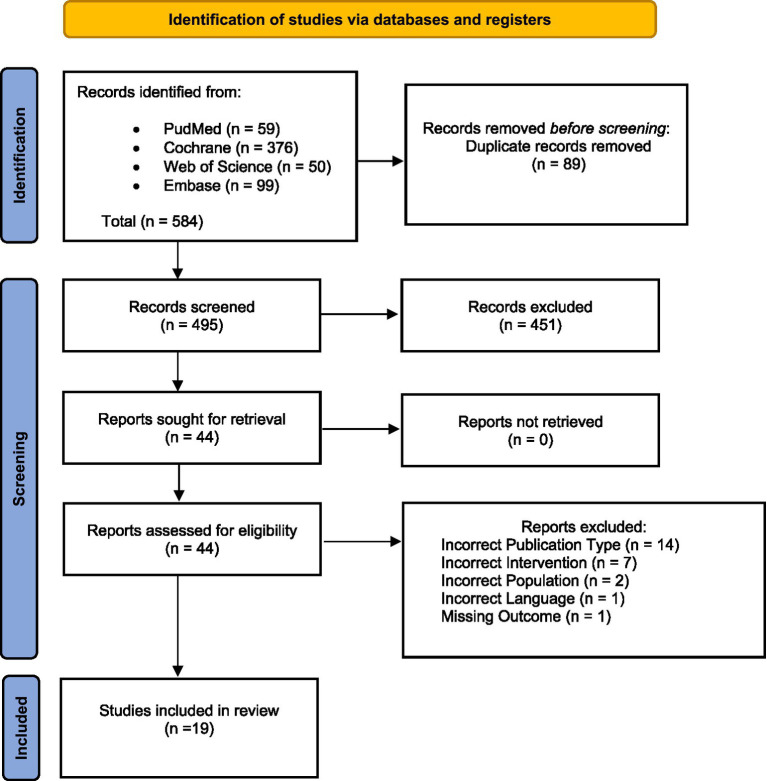
Flowchart of the study selection process.

A total of 584 records were identified through databases: PubMed (*n* = 59), Cochrane Library (*n* = 376), Web of Science (*n* = 50), and Embase (*n* = 99). After removing 89 duplicate records, 495 records remained for screening. Out of 495 records screened, 451 were excluded based on title and abstract. The remaining 44 full-text articles were assessed for eligibility. No reports were excluded due to retrieval issues. Upon full-text review, 25 reports were excluded for the following reasons: incorrect publication type (*n* = 14), incorrect intervention (*n* = 7), wrong population (*n* = 2), language issues (*n* = 1), and missing outcome data (*n* = 1). Ultimately, 19 studies met the inclusion criteria and were included in the final review (Figure 1).

### 3.2 Characteristics of source evidence and results

See Tables 1–3.

**Table 1:** Dance therapy intervention.

Authors	Year	Study design	Aim	Population	Intervention	Technology	Dose	Outcomes	Key findings
Parkinson									
Bevilacqua R. et al. (27)	2025	Technical feasibility pilot study	Assess the usefulness of a dance-based rehabilitation program enriched by artificial intelligence–based exergames and contextual robotic assistance	Parkinson’s Disease (*n* = 20)	Irish Dance via AI-enhanced exergames	SI-Robotics platform Wearable sensors and 3D cameras Robot Operating System (ROS)	16 sessions of 50 min (2 sessions per week, for 8 wks), involving 2 patients at a time.	UPDRS-III, SPPB, TUG, 6MWT, POMA (Total, Gait, Balance), SF-12, FES-I, UTAUT subscales	Significant improvements in motor function balance, and gait; Modest but significant improvement in physical performance; Positive feedback from users on system usability, enjoyment, and trust.
Tunur et al. (25)	2020	Pilot study	To assess the feasibility, safety, and acceptability of augmented reality dance therapy	Parkinson’s Disease (*n* = 14)	Dance via “Moving Through Glass” (MTG) app on Google Glass	Augmented reality (Google Glass)	three modules a day, every day, for 3 weeks	Feasibility included recruitment, retention, intervention adherence and safety. Secondary Mini-BESTest, 1-leg stance, TUG and Dual Task, Balance Confidence Scale, BDI, PD-QoL	Recruitment rate was 50%, retention rate was 100%, and adherence to usage was 95%. The Intervention was safe and accepted by participants. They improved mobility under cognitive load. No significant changes in balance, mood, or QoL.
Stroke									
Subramaniam & Bhatt (26)	2019	Preliminary study	To evaluate the effects of dance exergaming on upper limb rehabilitation and fall risk	Community-dwelling chronic stroke survivors (*n* = 13)	Dance exergaming to improve upper limb function and balance	Exergaming platform- Kinect dance game “Just Dance 3.”	10 songs 5session/week (for 2 weeks), 12 songs for 3 session (2 weeks), 14 songs for 2 session (remaining 2 weeks); total 20 sessions	Functional Stand Reaching test, dance movement test, ABC, TUG, ADL, EMG analysis, kinematics data	Motor function improved, fall risk reduced. Promising for chronic stroke rehab.

MDS-UPDRS, movement disorders society unified Parkinson disease rating scale; SPPB, short physical performance battery; 6MWT, six minute walking test; POMA, performance-oriented mobility assessment; FES-I, falls efficacy scale-international; UTAUT, unified theory of acceptance and use of technology; HADS, the hospital anxiety and depression scale; SF-12, 12-item short-form health survey; ABC, activities-specific balance confidence scale; FTSTS, five times sit to stand test; TUG, time up and go; FAC, functional ambulation categories; Mini-BESTest, mini balance evaluation system test; BDI, beck depression inventory; PD-QoL, Parkinson disease quality of life scale.

**Table 2:** Music therapy intervention.

Authors	Year	Study design	Aim	Population	Intervention	Technology	Dose	Outcomes	Key findings
Parkinson									
Impellizzeri F. et al. (28)	2024	Single-blind, quasi-randomized controlled trial	Assess whether coupling Neurologic Music Therapy (NMT) with immersive Virtual Reality (VR) using the CAREN platform enhances cognitive function	Parkinson’s Disease (*n* = 40)	Rhythmic Auditory Stimulation (RAS) and Therapeutic Instrumental Music Performance (TIMP)	CAREN platform of immersive Virtual Reality	8 weeks Sessions: 45 min, 3 times per week	MoCa, FAB, Stroop Test, Visual Search HAM-D	CAREN platform improves cognitive functions in PD.
Dauvergne et al. (31)	2018	Prospective single-arm pilot intervention study	To evaluate the usability, adherence, and effects of a music-based serious game on rhythmic skills in PD.	Parkinson’s Disease (*n* = 16)	Home-based rhythmic training using ‘Rhythm Workers’ serious game on a tablet	Tablet-based serious game (at home)	30 min/session for 3 sessions/week for 6 weeks	Usability, adherence, SEQ, BAT rhythmic skill scores	This pilot study shows that the Rhythm Workers game is well-suited for at-home rehabilitation of rhythmic skills in PD, with initial evidence of improved rhythm perception.
Stroke									
Segura et al. (30)	2021	Pilot Feasibility Study	To test the feasibility and usability of an app delivering home-based enriched Music-supported Therapy (eMST)	chronic stroke (*n* = 5)	tablet app, MIDI-piano, and percussion instruments; gamification, progress monitoring, and personalization features included	Tablet app	1 h/session 3 sessions/week for 10 weeks	Upper limb motor function (ARAT, FMA, BBT, NHPT, CAHAI), piano performance (speed, force tapping), usability (SUS)/feseability (interview); Adherence/Safety	The intervention was feasible and safe; improvements observed in motor function and piano performance; eMST showed promise as a telerehabilitation tool for stroke recovery.
Chang W. C. et al. (35)	2019	Pilot study	To analyze EEG patterns during mixed-reality music therapy for lower limb rehabilitation	stroke (*n* = 2)	MR music exercise presents holographic rhythm-game content. Participants step to music beats to hit targets.	Mixed-reality system (Hololens), IMU sensors	One single session (presence vs. absence of MR music)	Gait Kinematic outcome, EEG analysis	The patient’s motor function is significantly activating when wearing the MR system during the rehabilitation task. Flexion angle of the hemiplegic knee during walking was significantly improved when following the tempo of the MR music content.
Baka et al. (29)	2018	Preliminary qualitative clinical study	To explore virtual reality rehabilitation combined with neurologic music therapy	Stroke (*n* = 3)	In the VR system, patients play virtual instruments by following the movements of a friendly alien guide.	Virtual reality system (Oculus Rift and LeapMotion Controller)	15–20 min/session for 2 times/week for 2 weeks	Kinematics data Self efficacy	This system has enabled patients to enhance their motor skills more quickly, effectively, and with greater motivation.
Zondervan et al. (41)	2016	RCT	To assess the feasibility and efficacy of MusicGlove therapy for home-based hand rehabilitation.	Chronic stroke (*n* = 17) experimental group (*n* = 9); conventionaltherapy (*n* = 8)	MusicGlove is a device that guides users to perform hand grips in time with music—similar to Guitar Hero—using visual and audio cues to improve movement accuracy	Sensorized glove with game interface (home use)	3 h/week for 3 sessions/week for 3 weeks	Fugl-Meyer UE, MAL, NHPT, BBT, ARAT	MusicGlove is an effective home therapy tool. While primary outcomes were comparable to conventional therapy, MusicGlove led to significantly better results in two secondary measures: MAL Amount of Use and Quality of Movement.
Trobia et al. (36)	2011	Pilot feasibility study	To assess the feasibility of combining music and VR for supporting mental practice.	Chronic stroke (*n* = 2)	The upper limb training included VR-guided motor imagery with music during hospital sessions and daily home practice	VR Mirror system with DVD-based home training	4 weeks, 3 sessions/week + daily home	ARAT, Fugl-Meyer UE, VMIQ, MBEA, diary logs	Music and VR can be feasibly integrated into mental practice for stroke rehabilitation. Two patients showed motor and ADL improvements after 8 weeks.
Friedman et al. (42)	2011	Single-session experimental study	To assess the feasibility, effectiveness, and motivation associated with MusicGlove.	Chronic stroke (*n* = 10)	Single-session training with MusicGlove using music to guide gripping tasks	Sensorized glove + modified rhythm video game	Single session; 6 trials of ~3 min each (with and without music)	Box and Blocks, % of notes hit, note timing, motivation scale	Music feedback was found to be a motivating tool for repetitive hand grip training, with music significantly enhancing performance and motivation.
Others									
Jeong E. et al. (37)	2024	Single-blind randomized crossover study	Explore the effectiveness of virtual reality–based music attention training (VR-MAT) on cognitive function	Patients with acquired brain injury (*n* = 24)	Music therapy via rhythm-based, interactive virtual reality drumming tasks	Non-immersive virtual reality (VR) Computer-based rhythmic drumming game developed in Unity Drums connected via Android PC	8 weeks total Sessions: 30 min/day, 5 days/week	TMT-B&W-B and B–A (CDR-SB) (MMSE) CANTAB: SWM; AST SST VR-MAT accuracy (task-level performance)	VR-MAT was more effective for executive functions and attention switching.
Tamplin et al. (38)	2020	Development and feasibility study	To test the feasibility of group singing therapy via virtual reality for people with spinal cord injury	Patients with spinal cord injury (*n* = 6)	Group singing in online VR environment	Online virtual reality platform	Not specified	SUS, QUEST, PIADS + interviews	Usable and acceptable; supports social engagement and therapeutic potential of VR singing.
Cunningham et al. (32)	2019	Mixed-methods cohort study	To assess wellbeing outcomes from song-task association and musical reminiscence techniques in people with dementia	Individuals with dementia (*n* = 14)	Memory Tracks is an app that uses familiar songs to support daily tasks, helping with memory recall and reducing agitation	Mobile app	Every day in different time of the day in specific task	QoL-AD, SAM Scale, interviews with care staff	Mood, engagement, and wellbeing improved. Mobile music apps support dementia care.

MoCA, Montreal Cognitive Assessment; FAB, Frontal Assessment Battery; Stroop Test, Stroop Color and Word Test; Visual Search, Visual Search Test; HAM-D, Hamilton Rating Scale for Depression; UTAUT, Unified Theory of Acceptance and Use of Technology; SPPB, Short Physical Performance Battery; POMA, Performance-Oriented Mobility Assessment; FES-I, Falls Efficacy Scale-International; SEQ, Suitability Evaluation Questionnaire; BAT, Beat Alignment Test; ARAT, Action Research Arm Test; FMA, Fugl-Meyer Assessment (Upper Extremity); BBT, Box and Blocks Test; NHPT, Nine Hole Peg Test; CAHAI, Chedoke Arm and Hand Activity Inventory; MAL, Motor Activity Log; VMIQ, Vividness of Movement Imagery Questionnaire; MBEA, Montreal Battery of Evaluation of Amusia; TMT-B&W, Trail Making Test – Black & White version; CDR-SB, Clinical Dementia Rating – Sum of Boxes; MMSE, Mini-Mental State Examination; CANTAB, Cambridge Neuropsychological Test Automated Battery, comprensiva dei subtest; SWM, Spatial Working Memory; AST, Attention Switching Task; SST, Stop Signal Task; VR-MAT, Virtual Reality–based Music Attention Training; SUS, System Usability Scale; QUEST, Quebec User Evaluation of Satisfaction with Assistive Technology; PIADS, Psychosocial Impact of Assistive Devices Scale; QoL-AD, Quality of Life in Alzheimer’s Disease; SAM, Self-Assessment Manikin.

**Table 3:** (Visual) Art therapy intervention.

Authors	Year	Study design	Aim	Population	Intervention	Technology	Dose	Outcomes	Key findings
Stroke									
Tieri G. et al. (43)	2024	Single-blind randomized controlled trial	Assess the efficacy of a virtual reality (VR) art therapy protocol compared to conventional therapy	Stroke (*n* = 40)	Visual art therapy via virtual painting of masterpieces	Immersive virtual reality using Oculus Quest 2 headset and controllers	4 weeks; 3 sessions per week of 1 h each	BI; MMT; Ashworth scale; PRPS; fatigue	VR art therapy group had significantly better outcomes in Barthel Index, muscle strength and spasticity reduction. Effectiveness correlated with participation, satisfaction, and perceived utility.
De Giorgi R. et al. (47)	2023	Matched case –control study	To test the efficacy of a virtual art therapy (VAT) protocol based on the Michelangelo effect to improve upper limb recovery and daily living independence	Stroke (*n* = 20)	Visual art therapy using immersive VR to simulate paintings of famous masterpieces	Immersive virtual reality (Oculus Rift headset & controller)	1 month; 3 sessions per week of ~30 min	BI, (MMT), Ashworth Scale; PRPS, fatigue NRS	VAT group had significantly greater improvement in BI and pinch strength higher participation & lower fatigue
Alex et al. (39)	2021	Mixed methods: field study + technology probe	To explore traditional and VR-based art-making design implications for VR stroke rehab	Stroke (*n* = 14)	3-week field study of traditional art-making followed by experiential VR art sessions using Google Tilt Brush in a supportive studio environment	Virtual Reality (HTC Vive and Oculus Rift)	Traditional: weekly 2.5 h sessions for 3 weeks; VR: single 30–35 min session	Qualitative themes (engagement, control, physicality, expression)	VR art is immersive, fosters movement, but lacks tactile feedback and control; traditional art supports social, reflective, and intuitive engagement; both offer therapeutic potential
Iosa et al. (44)	2020	Experimental study	To examine the effects of art exposure on upper limb rehabilitation using virtual reality	stroke (*n* = 4) (2 art masterpiece stimuli vs. 2 control stimuli)	Visual art stimuli integrated in a VR-based motor task	Virtual reality system (Oculus rift) and an Oculus Controller joystick with virtual cavas	4 sessions of 10 stimuli over 8 days	FMA, Box & Block, 9-Hole Peg, USEQ, PRPS, NASA-TLX kinematics	Only the patients treated with artistic stimuli showed a significant reduction in erroneous movements performed orthogonally to the canvas, indicating improved motor accuracy. Moreover, patients reported high usability and satisfaction with the virtual reality system.
Others									
Worthen-Chaudhari et al. (40)	2013	Prospective feasibility study	To evaluate the feasibility of an interactive art-based feedback system for patients in acute neurorehabilitation.	Inpatients with various neurological conditions (*n* = 21)	A gyroscopic sensor captures movement, creating real-time abstract art on a computer screen. It offers instant visual feedback.	Motion sensors + interactive generative art software	The technology was used within 1 to 7 therapy sessions per participant.	FIM, therapy documentation, qualitative feedback from therapists	Interactive arts technology proved feasible and engaging in neurorehabilitation, supporting motor tasks and motivating patients, including those with low cognitive function. It shows promise for in-clinic and home use.

### 3.3 Synthesis of results

The included studies were grouped according to the type of art therapy intervention applied in virtual or technology-assisted formats, specifically: dance therapy, music therapy, and visual art therapy. A summary table was developed to consolidate key characteristics such as study design, aims, population, type of intervention, technology used, dosage, outcomes measured, and main findings.

#### 3.3.1 What are the main art therapy techniques and technologies used in neurorehabilitation?

Three main categories of virtual art therapy techniques emerged from the included studies: dance therapy, music therapy, and visual art therapy. Each demonstrated potential for enhancing neurorehabilitation across various neurological conditions including Parkinson’s disease (PD), stroke, acquired brain injury (ABI), dementia, and spinal cord injury.

***Dance Therapy***: Dance-based interventions often leveraged motion and rhythm to engage patients in rehabilitation tasks. Technologies used included:

- Augmented reality (AR), such as the *Moving Through Glass* app on Google Glass (25).
- Exergaming platforms, like Kinect’s *Just Dance 3* (26).
- AI-enhanced dance systems, like Irish dance integrated with robotic assistance (27).

These studies primarily targeted individuals with Parkinson’s disease and post-stroke motor deficits, showing positive effects on gait, balance, motor function during cognitive load and patient engagement.

***Music Therapy***: Music therapy represented the most frequently applied art form in virtual formats. Key techniques and technologies included:

- Immersive VR: Platform Coupled with Neurologic Music Therapy (NMT) to improve cognition in PD (28), throught oculus rift participants interact with virtual musical instruments (29) improving motor skills.
- Home-based music applications: enriched Music-supported Therapy via MIDI piano and percussion throught tablet app for stroke recovery improving motor function and piano performance (30), Tablet-based applications and serious games: *Rhythm Workers* to asses rhitmic skills on PD (31) and a Mobile app: *Memory Tracks* that is an app that uses familiar songs to support daily tasks in people with dementia (32).
- Sensorized devices: *MusicGlove*, guiding rhythm-based gripping tasks (33, 34) used also at home and able to improve the quality of movement.
- Mixed reality gait training: with Hololens and music exercise with holographic rhythm-game content for stroke rehabilitation. Participants step to music beats to hit targets, improving kinematics data (35).
- VR music gaming: VR-guided motor imagery with music (36) and virtual drumming for ABI in a rhythm-based attention training (VR-MAT) context (37).
- Group singing in VR: explored for its psychosocial effects in spinal cord injury (38).

***Visual Art Therapy***: Visual art therapy was most frequently applied to stroke rehabilitation. Techniques included:

- Immersive VR: painting of famous artworks: As in the studies by Tieri et al., De Giorgi et al. and Iosa et al. (43, 44, 47), which implemented the “Michelangelo effect”—a concept describing enhanced motor performance when replicating masterpieces and Creative VR expression: e.g., with *Google Tilt Brush* in traditional art-making (39).
- Motion-capture art generation: Worthen-Chaudhari et al. (40) used gyroscopic sensors to translate movement into abstract digital art on a computer screen, motivating participation among patients with various neurological conditions.

These interventions emphasized the value of artistic engagement in improving upper limb motor control, cognitive-emotional integration, and therapy participation.

#### 3.3.2 What are the positive and negative factors of art therapy applied with technologies?

**Positive *Factors***:

- High feasibility and acceptance across neurological populations (e.g., PD, stroke, dementia, ABI).
- Enhanced motivation and engagement, particularly in gamified or music-integrated systems and especially in immersive, expressive VR settings.
- Motor improvements: Gains observed in upper limb function, gait symmetry, grip strength, and balance.
- Cognitive and emotional benefits: Notably in attention, executive function, mood, and quality of life.
- High usability and adherence: Particularly in home-based formats with minimal external support.

***Negative or Limiting Factors***:

- Lack of tactile feedback: VR art-making lacks the physical texture of traditional materials.
- Limited long-term efficacy data: Many studies were pilots or single-session experiments.
- Adherence challenges: Especially in unsupervised or cognitively impaired populations.
- Small sample sizes and heterogeneous outcome measures, which reduce generalizability and limit comparative analyses across studies.

#### 3.3.3 What are the primary outcomes studied in the research on virtual art therapy in neurorehabilitation?

Outcome measures varied based on intervention type and target population, including:

- Motor Function: Fugl-Meyer Assessment, Action Research Arm Test (ARAT), Box and Blocks Test (BBT), Nine Hole Peg Test (NHPT), gait kinematics.
- Balance and Mobility: Timed Up and Go (TUG), Mini-BESTest, One-Leg Stance Test.
- Cognitive Function: Montreal Cognitive Assessment (MoCA), Frontal Assessment Battery (FAB), Stroop Test, CANTAB tasks, Trail Making Test (TMT).
- Mood and Quality of Life: Beck Depression Inventory (BDI), Hospital Anxiety and Depression Scale (HADS), PD-QoL, QoL-AD.
- Engagement and Usability: System Usability Scale (SUS), Suitability Evaluation Questionnaire (SEQ), qualitative interviews, diaries.
- Participation and Self-Efficacy: Pittsburgh Rehabilitation Participation Scale (PRPS), user satisfaction scores, Functional Independence Measure (FIM).

Overall, most studies demonstrated positive changes in targeted outcomes, especially in motor function, engagement, cognitive attention, and therapy satisfaction.

## 4 Discussion

Integrating the arts into physical therapy enhances not only psychomotor abilities, but also emotional and cognitive outcomes (41), for example, art therapy led to measurable gains in visual-cognitive tasks, eye-tracking performance, and overall motor function in Parkinson’s disease. These clinical improvements were paralleled by increased resting-state connectivity in visual cortical networks, indicating adaptive brain reorganization (4). This scoping review explored the integration of digital and immersive technologies into art therapy interventions aimed at neurorehabilitation. The findings demonstrate a growing interest in leveraging virtual environments, motion-tracking devices, and interactive software to deliver dance, music, and visual art therapies to individuals with neurological conditions such as stroke, Parkinson’s disease, acquired brain injury (ABI), dementia, and spinal cord injury.

Dance therapy supported by technology, such as the Moving Thought Glass (MTG) modules on Google Glass, can be safely implemented at home in people with Parkinson’s disease, with no reported falls. This approach showed improvements in dual-task cost, indicating a positive effect on complex mobility management (25). Integrating dance therapy into an exergame (a video game that requires physical movement) uses augmented reality to deliver guided exercises, enhancing engagement and motor aspects in both Parkinson’s and stroke populations (26, 27).

Among the three art modalities, music therapy appeared as the most widely adopted, likely due to its adaptability to digital formats and its strong theoretical links to motor and cognitive rehabilitation. Neurologic Music Therapy (NMT) represents a structured, evidence-based approach that utilizes the perception and production of music to enhance motor, cognitive, and emotional functions in individuals with neurological disorders (28, 42). When integrated with immersive Virtual Reality (VR), as demonstrated in the study by Impellizzeri et al., NMT becomes a fully engaging, 360-degree intervention. The CAREN platform, which emerges rhythmic auditory stimulation with interactive virtual environments, has shown significant improvements in cognitive domains such as executive function, attention, and processing speed in patients with Parkinson’s disease (28). This highlights the synergistic effect of music-based interventions and immersive technology in enhancing neuroplasticity.

Wearable systems, such as the MusicGlove, offer a complementary yet distinct advantage for home-based rehabilitation. These sensorized gloves transform therapeutic hand exercises into engaging, music-guided tasks through a “Guitar Hero” like interface. Studies (41, 42) indicate that such exergames not only improve motor function but also significantly boost user motivation and adherence, making them particularly suitable for chronic stroke patients engaging in long-term therapy. Similarly, *Rhythm Workers*, a tablet-based serious game designed for individuals with Parkinson’s disease, demonstrated promising results in enhancing rhythmic skills and user engagement in a home-based setting (31). Serious games (i.e., digital games designed for therapeutic purposes) facilitate motor learning, neuroplasticity, and active engagement by incorporating goal-directed, repetitive, and multisensory tasks. They enhance attention and motivation, provide real-time feedback, and allow for progress tracking and task personalization, all of which are critical components for effective neurorehabilitation (18, 19).

Supporting this, Chang et al. (35) used EEG analysis during mixed-reality music therapy for post-stroke lower limb rehabilitation and demonstrated significant activation of motor areas in the brain. Their findings also showed that providing real-time feedback through rhythmic, interactive tasks improved motor performance, consistent with established evidence that feedback enhances motor learning and recovery.

Furthermore, music-based rehabilitation approaches are being successfully extended beyond Parkinson’s and stroke. For example, group singing therapy in virtual reality environments has been shown to promote social engagement and psychological wellbeing in individuals with spinal cord injury (38). In the context of dementia, mobile applications such as *Memory Tracks* use familiar songs associated with daily tasks to stimulate memory recall and reduce agitation, with promising effects on mood and quality of life (32).

Music and VR have also been effectively combined with mental practice strategies, as seen in the pilot study by Trobia et al. (36), where patients engaged in motor imagery enhanced by music and virtual environments. This combination facilitates motor recovery by stimulating the brain areas involved in movement planning, even in the absence of physical execution, providing a valuable complement to active rehabilitation.

Overall, the integration of NMT and music-based interventions into advanced technologies—ranging from immersive VR to wearable exergames and mobile apps—offers a versatile and scalable model for neurorehabilitation. These approaches not only support cognitive and motor recovery but also enhance patient engagement, emotional wellbeing, and adherence across a wide range of neurological conditions.

Recent advances in neurorehabilitation have increasingly explored the integration of immersive technologies with creative therapies, such as virtual reality (VR)-based art therapy, to enhance motor recovery and participation in patients with neurological disorders, particularly stroke. Studies by Tieri et al. and De Giorgi et al. (43, 47) demonstrate that VR art therapy—where patients engage in virtual painting of masterpieces using headsets like Oculus Quest 2 or Oculus Rift—can significantly improve upper limb motor function, independence in daily living (Barthel Index), muscle strength, and reduce spasticity. These positive outcomes are correlated with higher patient participation, satisfaction, and perceived utility, highlighting the motivational benefits of immersive creative engagement.

A key concept emerging from these studies is the Michelangelo Effect, as described by Tieri et al. (43). This phenomenon refers to the improvement in motor performance and reduction in perceived fatigue observed when individuals interact with artistic content in immersive environments. Specifically, when patients engage in goal-directed movements—such as virtual painting—in the context of masterpieces, the esthetic and symbolic nature of the artwork appears to enhance motivation and provide a sense of purpose. This effect likely activates reward-related neural circuits, which may explain the increased engagement and reduced effort perception during rehabilitation tasks. The *Michelangelo Effect* thus offers a theoretical foundation for understanding why immersive art therapy is particularly motivating and effective, combining emotional engagement with motor and cognitive stimulation.

Indeed, Iosa et al. (44) provide evidence that exposure to visual art stimuli during VR-based motor tasks improves motor accuracy in post-stroke patients, further supporting the role of artistic content as a meaningful and engaging context for neurorehabilitation.

Similarly, qualitative work by Alex et al. (39) comparing traditional and VR-based art-making reveals that VR environments provide an immersive and physically engaging experience that fosters movement, though traditional art offers tactile feedback and social interaction. Both approaches show therapeutic potential, suggesting that combining or alternating modalities could optimize rehabilitation outcomes.

Beyond stroke, interactive art-based feedback systems employing motion sensors and generative art software (40) have shown feasibility and motivation-enhancing effects in acute neurorehabilitation, including patients with varous neurological conditions, demonstrating versatility for clinical and potentially home use. Interactive arts technology was successfully integrated into neurorehabilitation, enhancing both fine and gross motor tasks while engaging patients across ages, genders, and cognitive levels. It provided unique graphic-art feedback, promoting a flow-like state (40).

Moreover, by leveraging core principles of the arts—such as the induction of flow states, emotional resonance, and the embodied power of music and dance—technology-enhanced interventions can generate more meaningful and lasting therapeutic outcomes (15). When artistic expression is integrated with digital platforms, it not only amplifies emotional engagement and enjoyment but also activates motivation and reward circuits in the brain, promoting neuroplasticity. Additionally, the use of technology enables real-time, individualized feedback—an essential factor in motor learning and cognitive recovery (45). This synergy between artistic creativity and technological precision supports a more dynamic, adaptive, and motivating rehabilitation experience.

Finally, technologies in rehabilitation offer significant benefits for both patients and therapists. They enable consistent, extended training sessions while collecting objective data on patient progress. Automation allows multiple patients to be treated simultaneously and, via telerehabilitation, remotely, increasing access to care. For therapists, these technologies improve efficiency and optimize time with each patient, while for patients they enhance therapy availability and engagement through interactive and motivational tools (46).

### 4.1 Limitations

This scoping review presents several limitations that should be considered when interpreting the findings. First, the number of eligible studies included was limited (*n* = 19), with small sample sizes across most interventions, reducing the statistical power and generalizability of the results. Furthermore, the majority of studies were preliminary in nature—such as pilot or feasibility trials—with few randomized controlled trials (RCTs) available. This limits the strength of the evidence supporting the effectiveness of technology-assisted art therapy in neurorehabilitation.

Another limitation is the heterogeneity of study designs, populations, technologies, outcome measures, and therapeutic modalities, which made direct comparison across studies difficult. In many cases, outcome measures were not standardized, and follow-up periods were short or absent, restricting insights into long-term efficacy and sustainability of interventions.

Additionally, most studies lacked control groups, and blinding was rarely implemented, introducing potential bias. Technological limitations, such as lack of tactile feedback in virtual environments, were also identified as barriers to patient engagement, particularly in visual art therapies.

Lastly, language and publication restrictions (e.g., English-only, peer-reviewed journals) may have excluded relevant data from non-English, potentially affecting the comprehensiveness of the review.

### 4.2 Implications for future research and practice

The findings of this review highlight the therapeutic potential of technology-assisted art therapy in neurorehabilitation, while also pointing to clear areas for future research and refinement:

1. Standardized protocols: Future studies should define detailed intervention protocols, specifying session duration, frequency, type of art activity (e.g., VR painting, exergames, music therapy), and progression criteria. For example, a structured program could include 30-min VR painting sessions, three times per week, for 8–12 weeks, with adjustable difficulty levels.
2. Outcome measures: Research should incorporate multi-dimensional outcomes to capture motor, cognitive, emotional, and engagement effects.
3. Personalized rehabilitation: Interventions should be tailored to individual patient characteristics, including neurological condition, severity, age, cognitive status, and digital literacy. Adaptive technologies that adjust difficulty in real time based on patient performance can optimize engagement and therapeutic challenge.
4. Hybrid models: Combining immersive digital environments with tactile or physical interfaces—such as haptic gloves, robotic assistance, or real instruments—can integrate sensory feedback with motor practice, enhancing realism and engagement. For instance, VR painting programs coupled with haptic styluses can provide force feedback during brush strokes.
5. Long-term effects: Longitudinal studies should assess the sustainability of therapeutic gains at multiple follow-up points (e.g., 3, 6, 12 months), including motor, cognitive, and emotional outcomes, as well as adherence and engagement in home-based programs.
6. Diverse populations: Future research should include participants with a broad range of neurological conditions (e.g., stroke, Parkinson’s disease, ABI, dementia, spinal cord injury) and demographic variability (age, sex, cultural background) to determine cross-condition applicability and ensure generalizability.
7. Interdisciplinary collaboration: Effective development of technology-assisted art therapy requires collaboration among clinicians, engineers, designers, and patients. Patient-centered co-design can help ensure interventions are functional, meaningful, and motivating for users with neurological impairments.

## 5 Conclusion

Technology-enhanced art therapies, including dance, music, and visual art interventions delivered via virtual and digital platforms, are increasingly explored in neurorehabilitation. Current evidence indicates these approaches are feasible, engaging, and acceptable to patients. However, there is no high-quality evidence demonstrating their effectiveness in improving motor or non-motor outcomes in neurological disorders. Most studies are small, exploratory, or pilot trials, highlighting a critical need for well-powered randomized controlled trials with standardized outcome measures. Future research should also investigate long-term effects, personalized interventions, and application across diverse neurological populations to determine their true therapeutic potential.

## Footnotes

1
https://osf.io/fwtgk

## Appendix 1

**Table:**

Database	Final search phrase
Pudmed	(((((((((“Stroke”[Mesh]) OR “Parkinson Disease”[Mesh]) OR “Multiple Sclerosis”[Mesh]) OR “Spinal Cord Injuries”[Mesh]) OR “Alzheimer Disease”[Mesh]) OR “Neurodegenerative Diseases”[Mesh]) OR “Cerebrovascular Disorders”[Mesh]) OR “Brain Injuries”[Mesh]) OR “Parkinsonian Disorders”[Mesh]) OR “Amyotrophic Lateral Sclerosis”[Mesh] OR stroke OR “parkinson disease*” OR “multiple sclerosis” OR “spinal cord injur*” OR “Nervous system disease*” OR “Neurologic* diseases*” OR “Alzheimer disease*” OR “Neurodegenerative disease*” OR “Neurodegenerative disorder*” OR “Cerebrovascular disorder*” OR “Brain injur*” OR “Parkinsonian Disorder*” OR Parkinsonism OR “Amyotrophic lateral sclerosis”) AND ((“Art Therapy”[Mesh]) OR “Music Therapy”[Mesh] OR “Dance Therapy”[Mesh] OR Neuroaesthetics* OR Neuroesthetic* OR “Art therap*” OR “Music Therap*” OR “Dance therap*” OR “Performing art*” OR “Visual art*” OR “Art-based therap*” OR pottery OR “Digital art*” OR Poetry OR “Therapeutic art*” OR “Theather Therap*”) AND ((“Virtual Reality”[Mesh] OR “Virtual Reality Exposure Therapy”[Mesh] OR “Exergaming”[Mesh] OR “Biomedical Technology”[Mesh] OR “User-Computer Interface”[Mesh] OR “Augmented Reality”[Mesh] OR “Telerehabilitation”[Mesh]) OR “Virtual reality” OR “Virtual reality exposure therap*” OR Exergam* OR “Virtual environment*” OR “Virtual experience*” OR “Tilt brush” OR “Biomedical technolog*” OR “Digital rehabilitation” OR “User-computer interface” OR “Augmented reality” OR “Virtual rehabilitation” OR “Serious game*” OR Telerehabilitation OR “Mixed Reality”)
Web of Science	((ALL = (stroke OR “parkinson disease*” OR “multiple sclerosis” OR “spinal cord injur*” OR “Nervous system disease*” OR “Neurologic* diseases*” OR “Alzheimer disease*” OR “Neurodegenerative disease*” OR “Neurodegenerative Disorder*” OR “Neurologic Disorder*” OR “Cerebrovascular Disorder*” OR “Brain Injur*” OR “Parkinsonian disorder*” OR Parkinsonism OR “Amyotrophic lateral sclerosis”)) AND ALL = (Neuroaesthetics* OR Neuroesthetic* OR “Art therap*” OR “Music Therap*” OR “Dance therap*” OR “Performing art*” OR “Visual art*” OR “Art-based therap*” OR pottery OR “Digital art*” OR Poetry OR “Therapeutic art*” OR “Theather Therap*”)) AND ALL = (“Virtual reality” OR “Virtual reality exposure therap*” OR Exergam* OR “Virtual environment*” OR “Virtual experience*” OR “Tilt brush” OR “Biomedical technolog*” OR “Digital rehabilitation” OR “User-computer interface” OR “Augmented reality” OR “Virtual rehabilitation” OR “Serious game*” OR Telerehabilitation OR “Mixed Reality”)
